# Supplementary figures and images for: Investigating GERMs: How Genotype, Environment, and Rhizosphere Microbiome interactions underlie heat response in maize and sorghum
Source: bioRxiv. 2025 Dec 10:2025.12.10.693489. Preprint. [Version 1] doi: 10.64898/2025.12.10.693489 (PMC12707280; doi:10.64898/2025.12.10.693489)

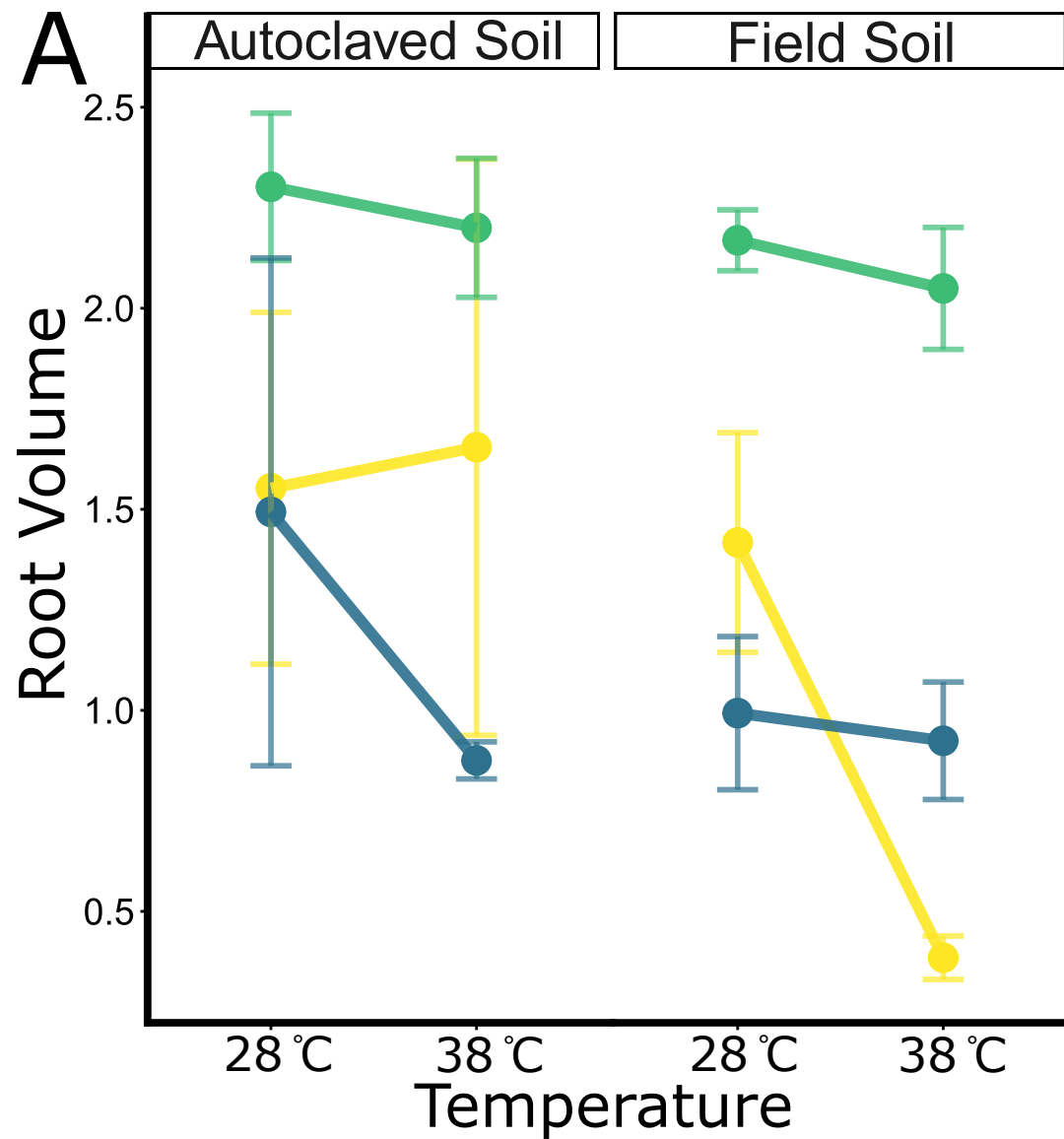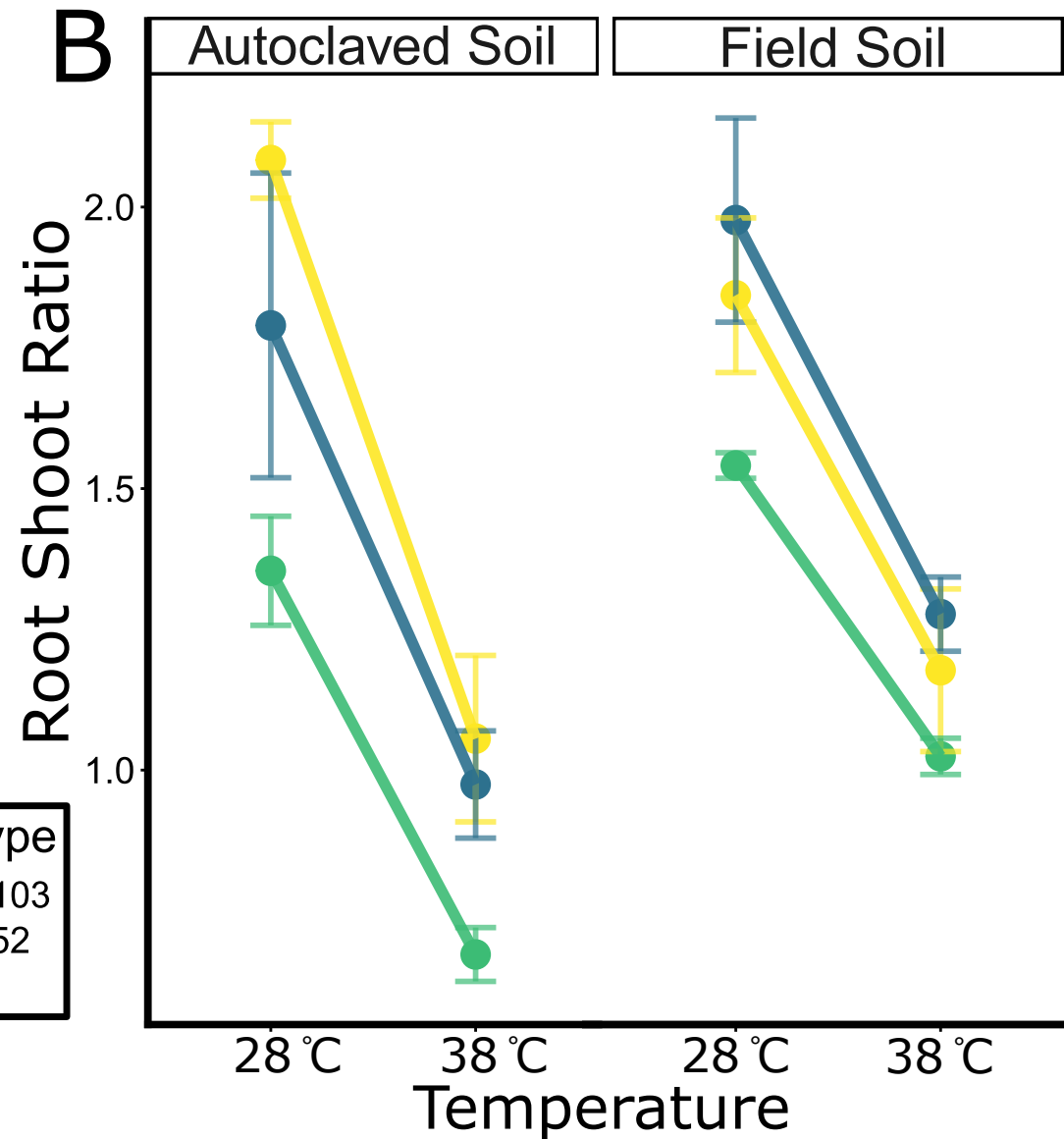

Supplement: Supplement 8 [file media-8.pdf]

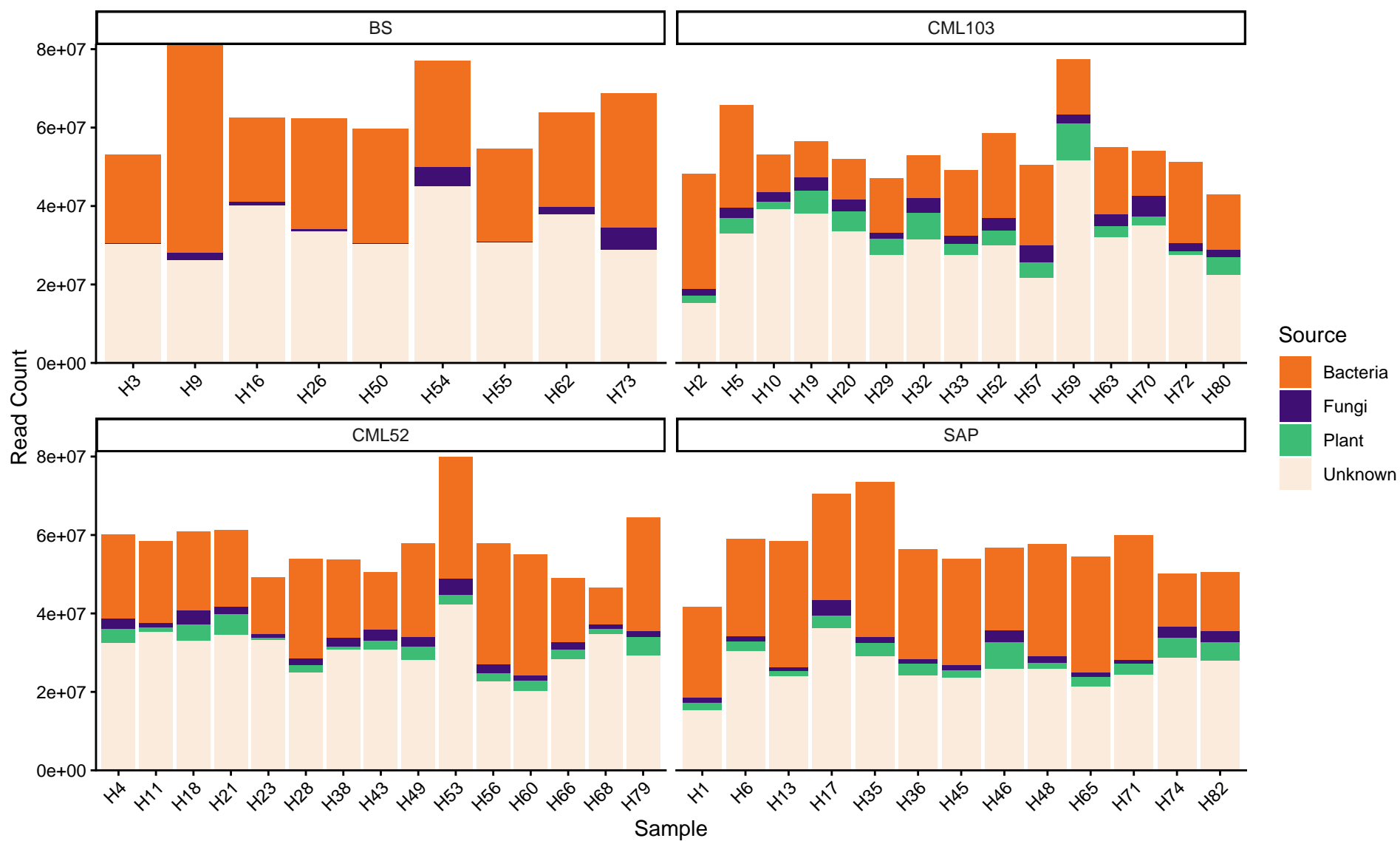

Supplement: Supplement 9 [file media-9.pdf]

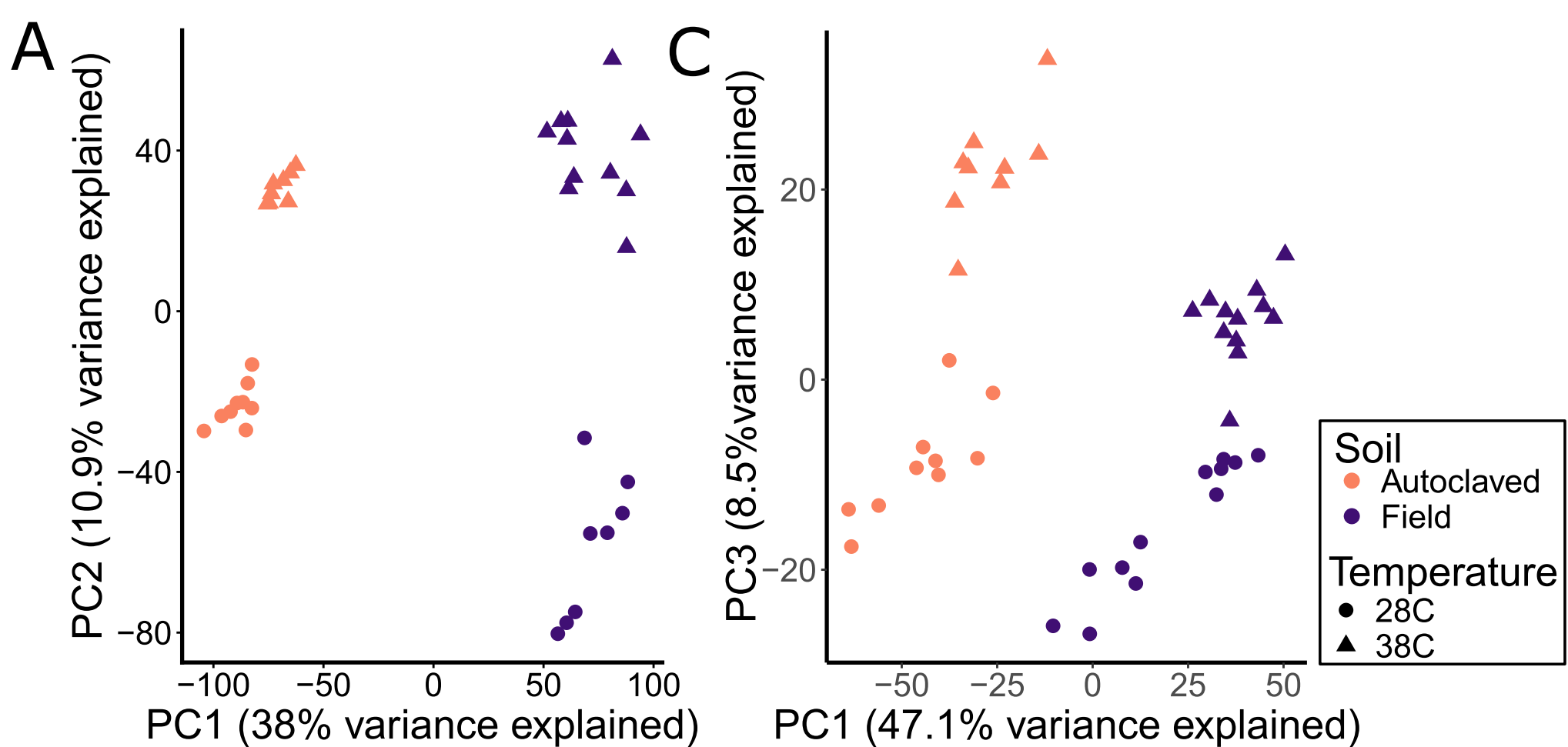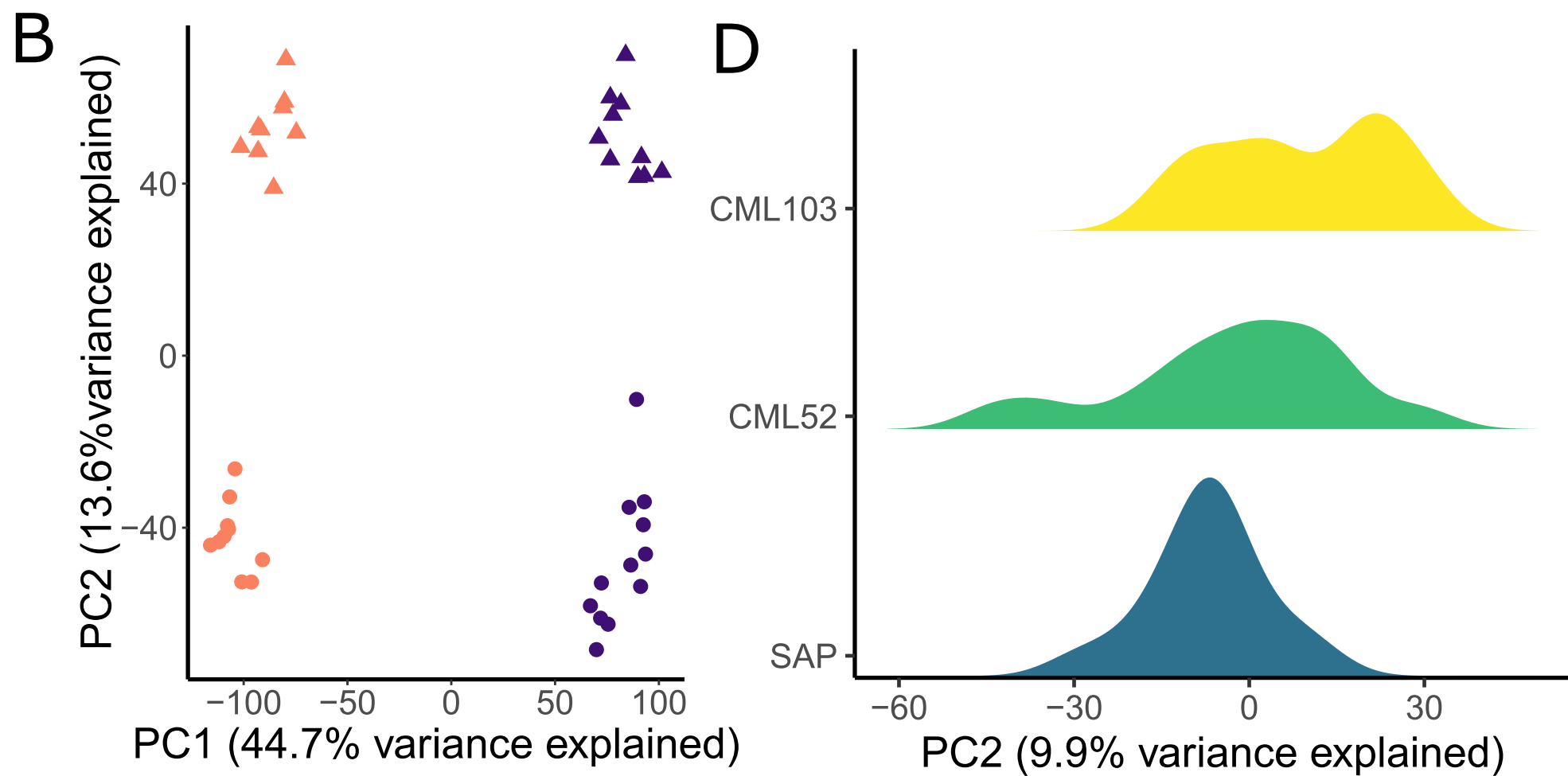

Supplement: Supplement 10 [file media-10.pdf]

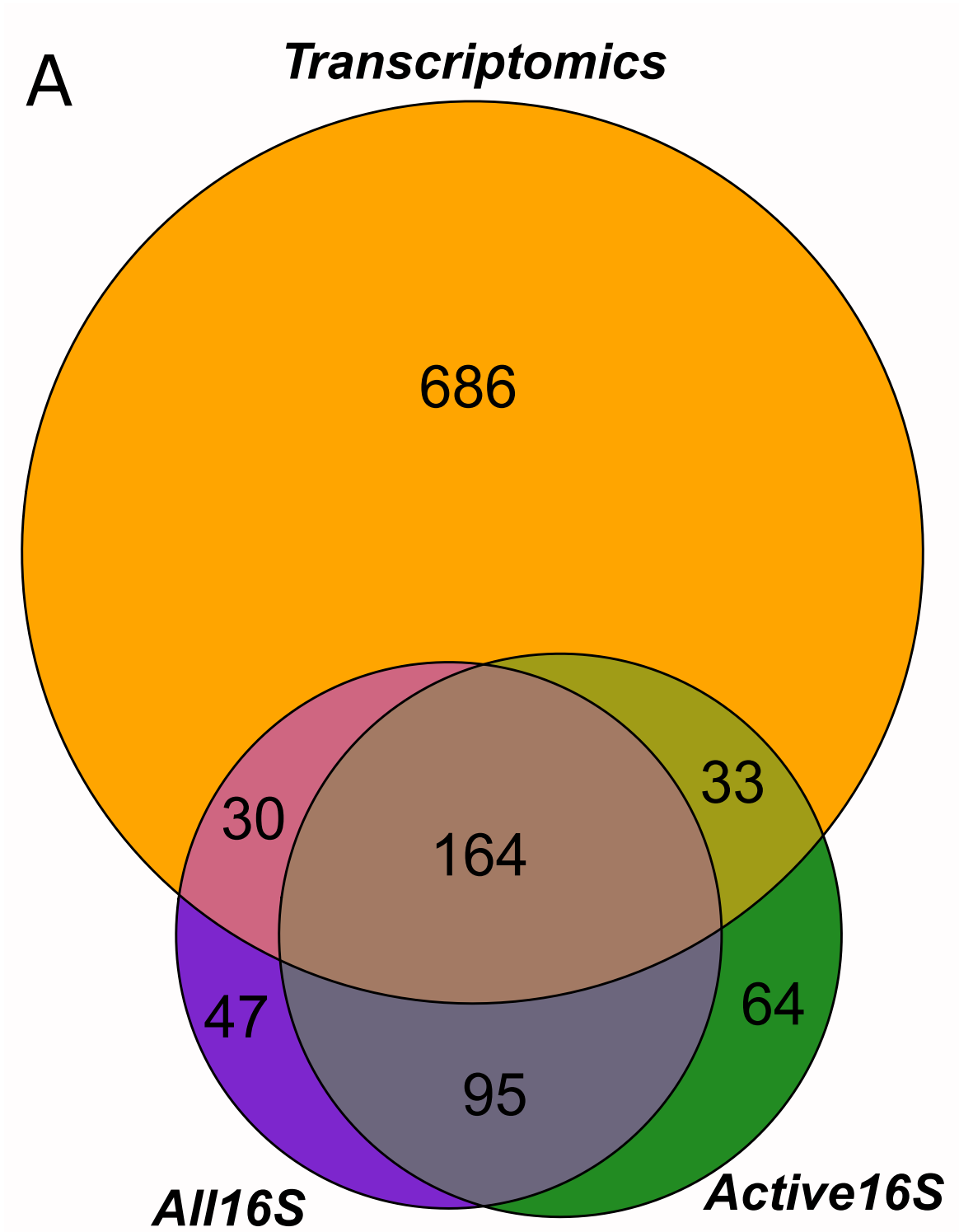

Overrepresented in:

- Transcriptomics
- Active 16S
- All 16S

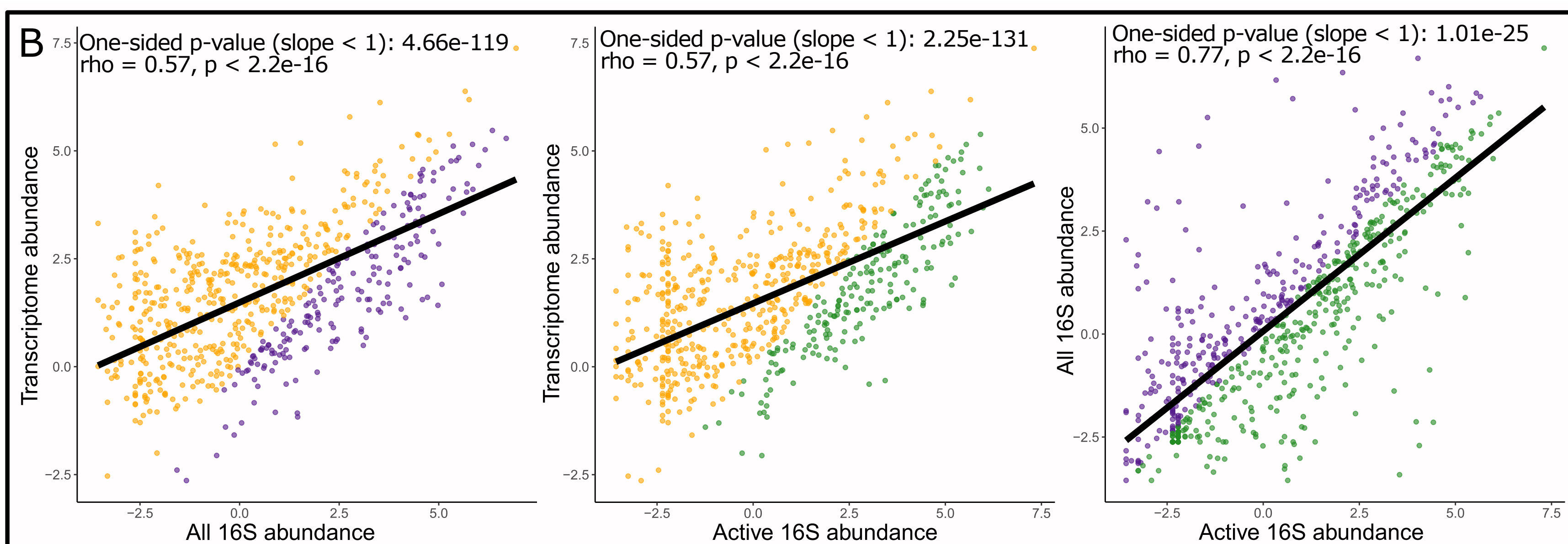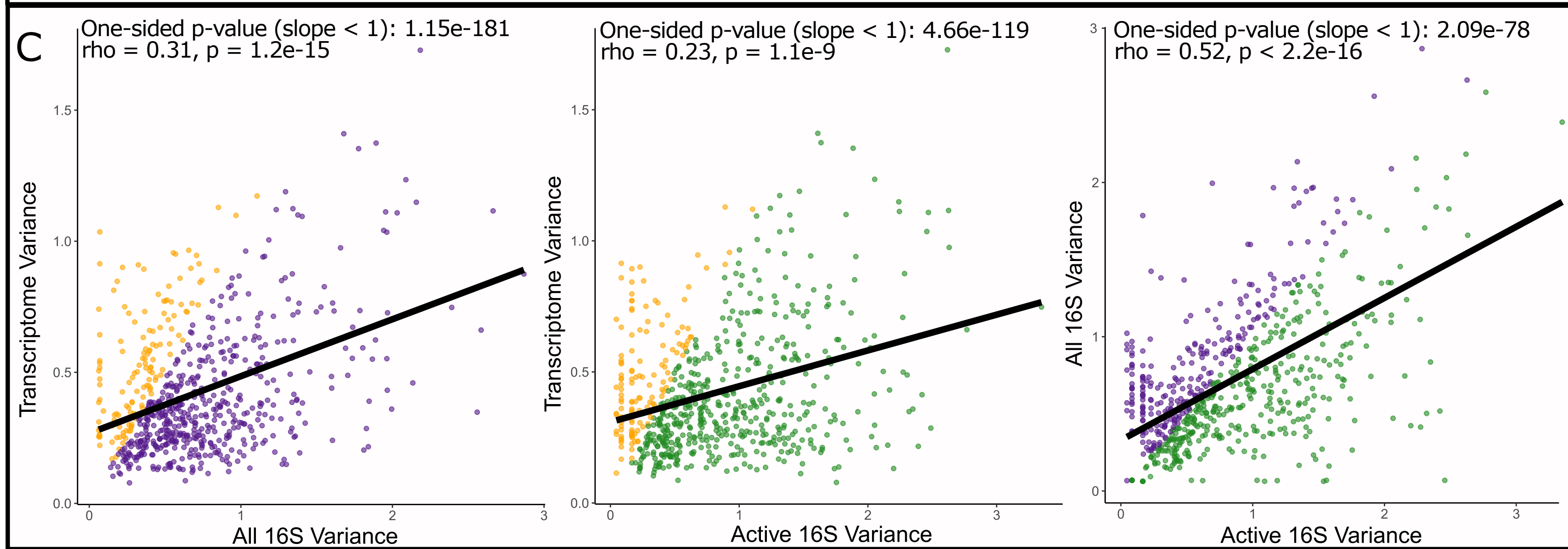

Supplement: Supplement 11 [file media-11.pdf]

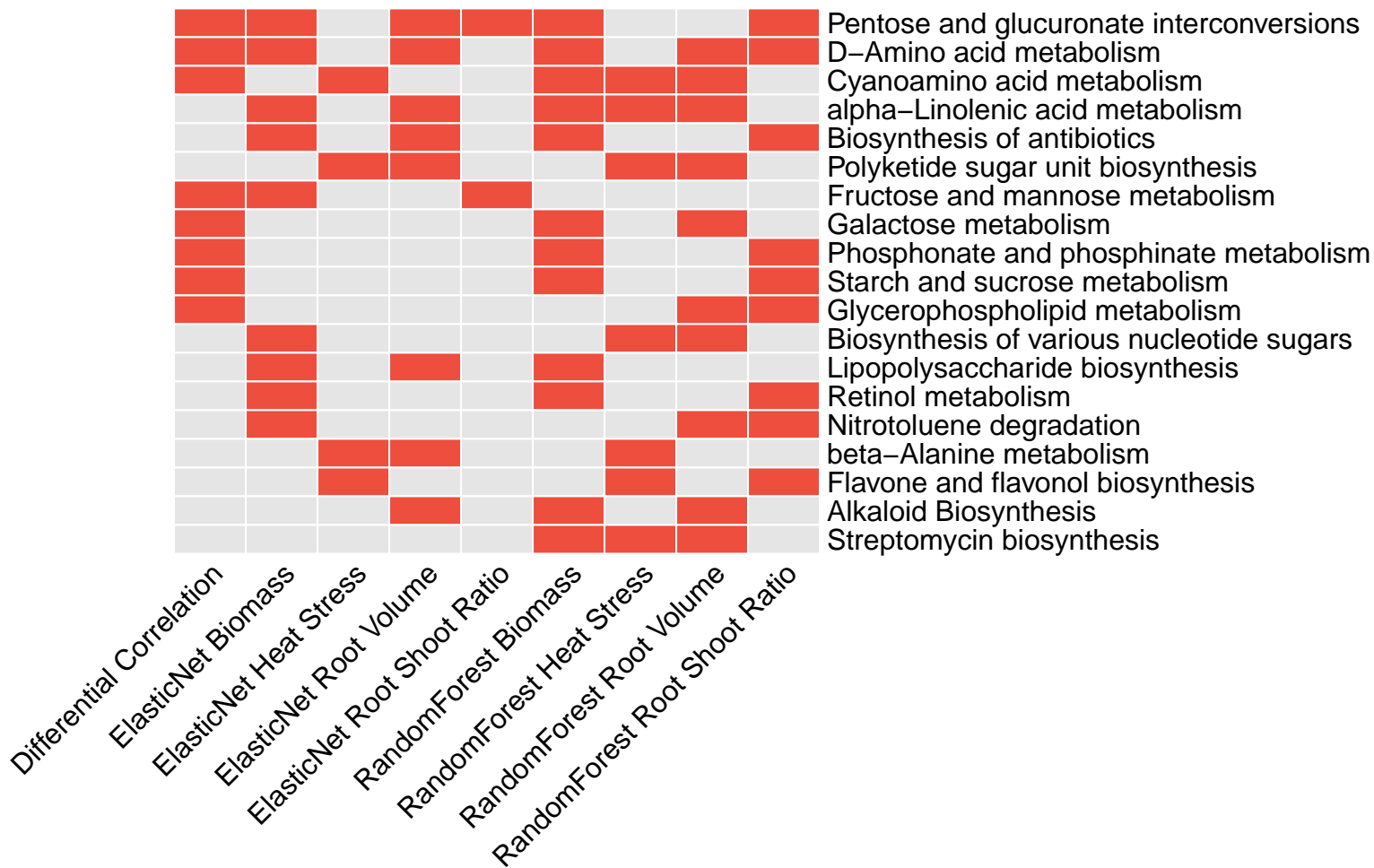

Supplement: Supplement 13 [file media-13.pdf]

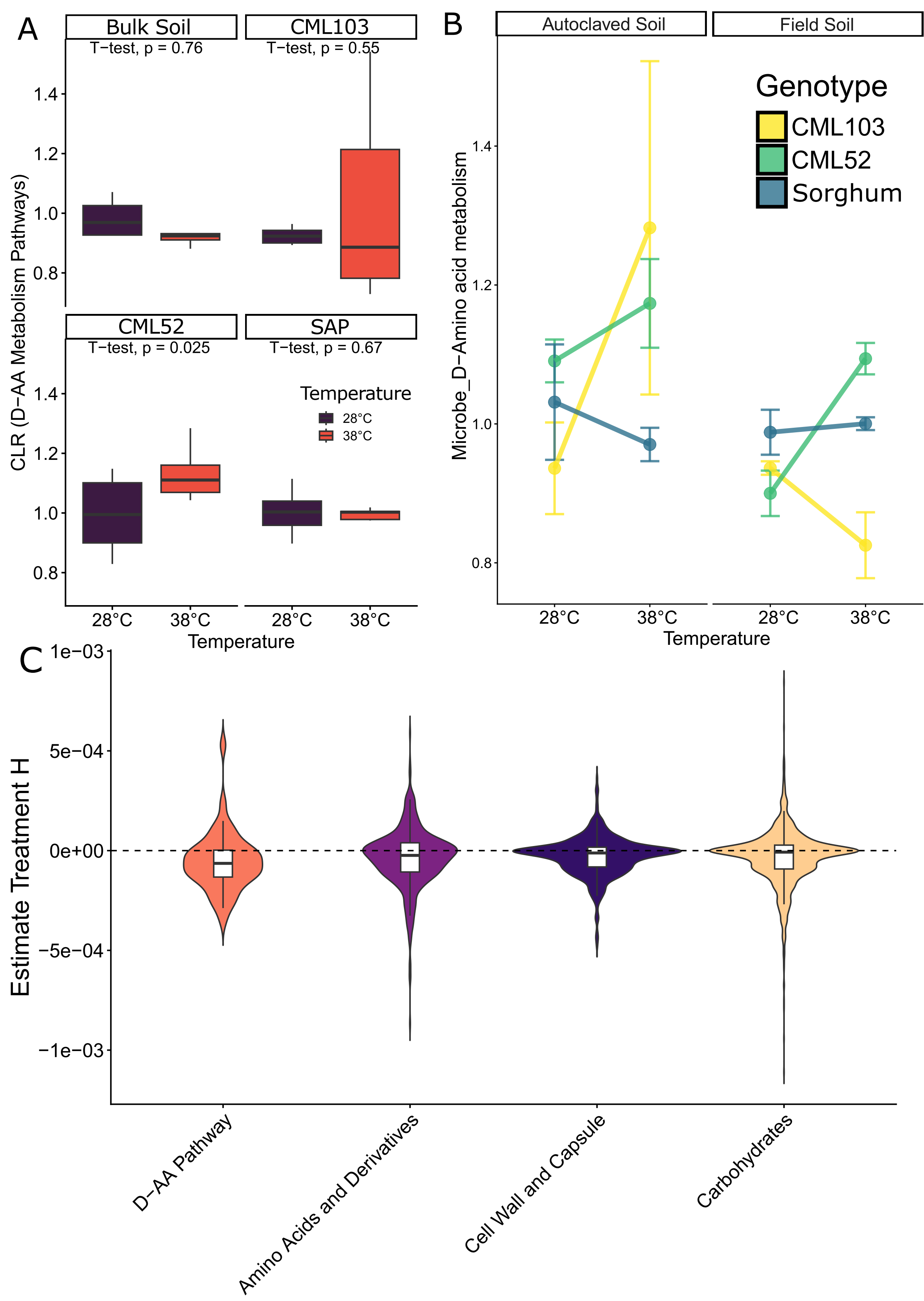

Supplement: Supplement 14 [file media-14.pdf]
