# Supplementary material for: Investigating GERMs: How Genotype, Environment, and Rhizosphere Microbiome interactions underlie heat response in maize and sorghum
Source: bioRxiv. 2025 Dec 10:2025.12.10.693489. Preprint. [Version 1] doi: 10.64898/2025.12.10.693489 (PMC12707280; doi:10.64898/2025.12.10.693489)
Supplement: Supplement 12 [file media-12.pdf]

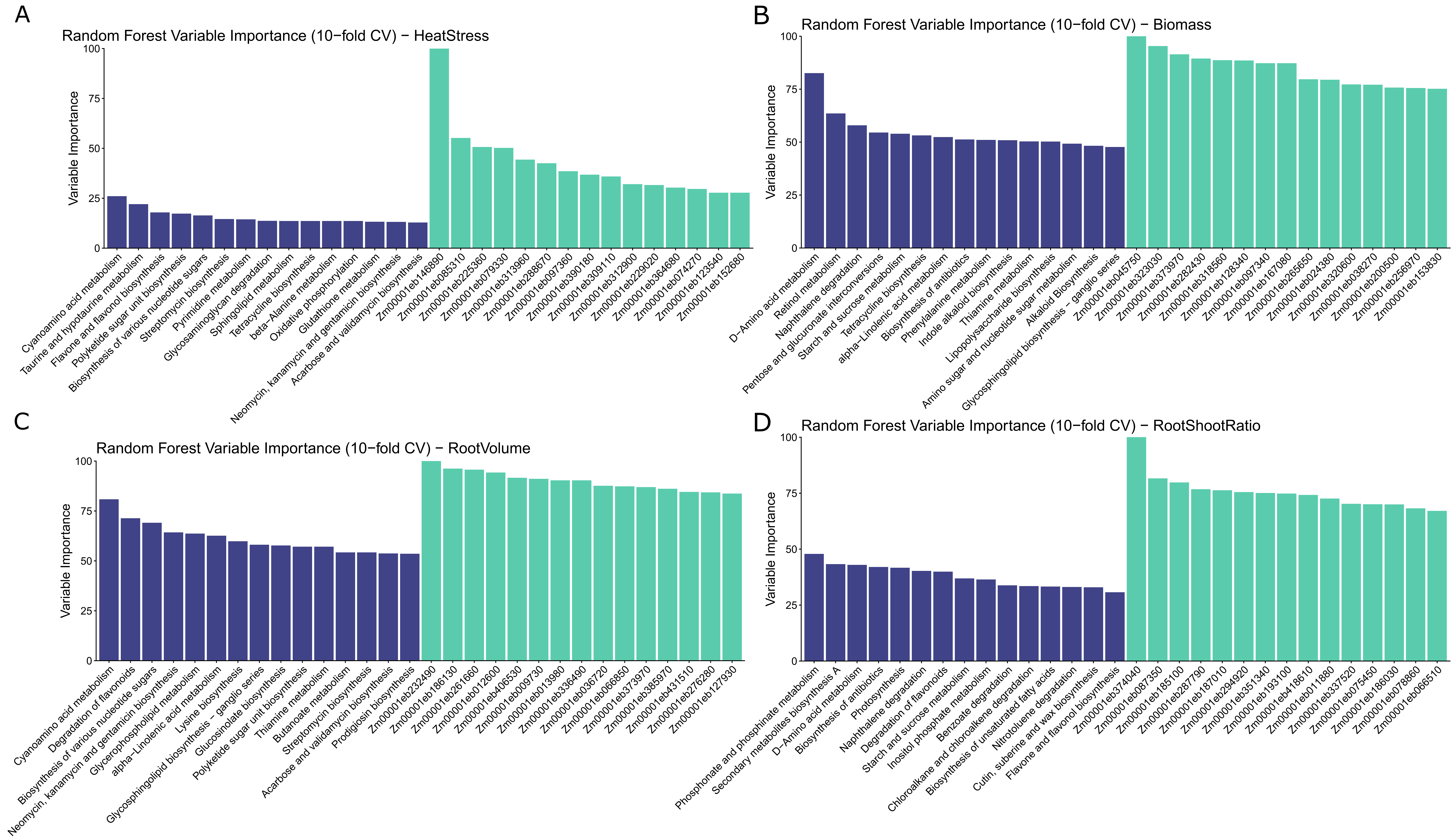
A
Random Forest Variable Importance (10−fold CV) − HeatStress
Variable Importance
0
25
50
75
100
Cyanoamino acid metabolism
Taurine and hypotaurine metabolism
Flavone and flavonol biosynthesis
Polyketide sugar unit biosynthesis
Biosynthesis of various nucleotide sugars
Streptomycin biosynthesis
Pyrimidine metabolism
Glycosaminoglycan degradation
Sphingolipid metabolism
Tetracycline biosynthesis
beta−Alanine metabolism
Oxidative phosphorylation
Glutathione metabolism
Neomycin, kanamycin and gentamicin biosynthesis
Acarbose and validamycin biosynthesis
Zm00001eb146890
Zm00001eb085310
Zm00001eb225360
Zm00001eb079330
Zm00001eb313960
Zm00001eb288670
Zm00001eb097360
Zm00001eb390180
Zm00001eb309110
Zm00001eb312900
Zm00001eb229020
Zm00001eb364680
Zm00001eb074270
Zm00001eb123540
Zm00001eb152680
B
Random Forest Variable Importance (10−fold CV) − Biomass
Variable Importance
0
25
50
75
100
D−Amino acid metabolism
Retinol metabolism
Naphthalene degradation
Pentose and glucuronate interconversions
Starch and sucrose metabolism
Tetracycline biosynthesis
alpha−Linolenic acid metabolism
Biosynthesis of antibiotics
Phenylalanine metabolism
Indole alkaloid biosynthesis
Thiamine metabolism
Lipopolysaccharide biosynthesis
Amino sugar and nucleotide sugar metabolism
Alkaloid Biosynthesis
Glycosphingolipid biosynthesis − ganglio series
Zm00001eb045750
Zm00001eb323030
Zm00001eb373970
Zm00001eb282430
Zm00001eb318560
Zm00001eb128340
Zm00001eb097340
Zm00001eb167080
Zm00001eb265650
Zm00001eb024380
Zm00001eb320600
Zm00001eb038270
Zm00001eb200500
Zm00001eb256970
Zm00001eb153830
C
Random Forest Variable Importance (10−fold CV) − RootVolume
Variable Importance
0
25
50
75
100
Cyanoamino acid metabolism
Degradation of flavonoids
Biosynthesis of various nucleotide sugars
Neomycin, kanamycin and gentamicin biosynthesis
Glycerophospholipid metabolism
alpha−Linolenic acid metabolism
Lysine biosynthesis
Glycosphingolipid biosynthesis − ganglio series
Glucosinolate biosynthesis
Polyketide sugar unit biosynthesis
Thiamine metabolism
Butanoate metabolism
Streptomycin biosynthesis
Acarbose and validamycin biosynthesis
Prodigiosin biosynthesis
Zm00001eb232490
Zm00001eb186130
Zm00001eb261660
Zm00001eb012600
Zm00001eb406530
Zm00001eb009730
Zm00001eb013980
Zm00001eb336490
Zm00001eb036720
Zm00001eb066850
Zm00001eb373970
Zm00001eb385970
Zm00001eb431510
Zm00001eb276280
Zm00001eb127930
D
Random Forest Variable Importance (10−fold CV) − RootShootRatio
Variable Importance
0
25
50
75
100
Phosphonate and phosphinate metabolism
Secondary metabolites biosynthesis A
D−Amino acid metabolism
Biosynthesis of antibiotics
Photosynthesis
Naphthalene degradation
Degradation of flavonoids
Starch and sucrose metabolism
Inositol phosphate metabolism
Benzoate degradation
Chloroalkane and chloroalkene degradation
Biosynthesis of unsaturated fatty acids
Nitrotoluene degradation
Cutin, suberine and wax biosynthesis
Flavone and flavonol biosynthesis
Zm00001eb374040
Zm00001eb087350
Zm00001eb185100
Zm00001eb287790
Zm00001eb187010
Zm00001eb294920
Zm00001eb351340
Zm00001eb193100
Zm00001eb418610
Zm00001eb011880
Zm00001eb337520
Zm00001eb075450
Zm00001eb186030
Zm00001eb078860
Zm00001eb066510
